# Supplementary figures and images for: Intraperitoneal Injection of the Porphyromonas gingivalis Outer Membrane Vesicle (OMV) Stimulated Expressions of Neuroinflammatory Markers and Histopathological Changes in the Brains of Adult Zebrafish
Source: Int J Mol Sci. 2024 Oct 14;25(20):11025. doi: 10.3390/ijms252011025 (PMC11506875; doi:10.3390/ijms252011025)

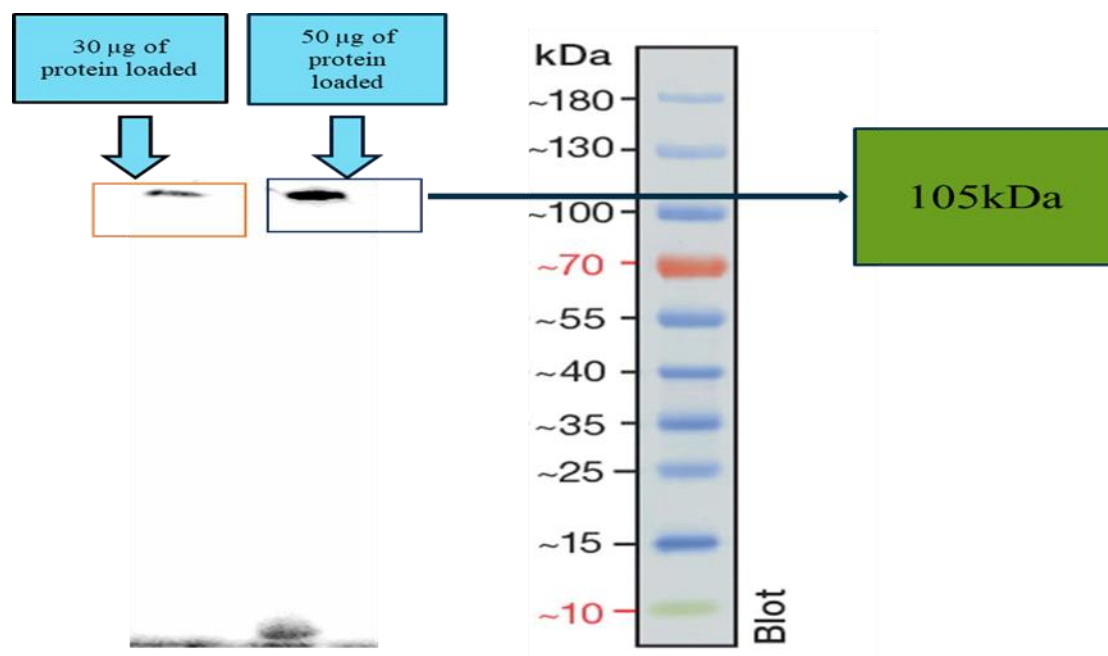

Figure S1 Western blot analysis showed the detection of gingipain.

Supplement: Supplementary file 1 [file ijms-25-11025-s001.zip › ijms-3196323-supplementary.pdf]
